# Resolving metal-molecule interfaces at single-molecule junctions

**DOI:** 10.1038/srep26606

**Published:** 2016-05-25

**Authors:** Yuki Komoto, Shintaro Fujii, Hisao Nakamura, Tomofumi Tada, Tomoaki Nishino, Manabu Kiguchi

**Affiliations:** 1 Department of Chemistry, Tokyo Institute of Technology O-okayama, Meguro-ku, Tokyo 152-8551, Japan; 2 National Institute of Advanced Industrial Science and Technology, Central 2, Umezono 1-1-1, Tsukuba, Ibaraki 305-8568, Japan; 3 Materials Research Center for Element Strategy, Tokyo Institute of Technology, 4259-S2-13 Nagatsuta-cho, Midori-ku, Yokohama 226-8503, Japan

## Abstract

Electronic and structural detail at the electrode-molecule interface have a significant influence on charge transport across molecular junctions. Despite the decisive role of the metal-molecule interface, a complete electronic and structural characterization of the interface remains a challenge. This is in no small part due to current experimental limitations. Here, we present a comprehensive approach to obtain a detailed description of the metal-molecule interface in single-molecule junctions, based on current-voltage (*I*-*V*) measurements. Contrary to conventional conductance studies, this *I*-*V* approach provides a correlated statistical description of both, the degree of electronic coupling across the metal-molecule interface, and the energy alignment between the conduction orbital and the Fermi level of the electrode. This exhaustive statistical approach was employed to study single-molecule junctions of 1,4-benzenediamine (BDA), 1,4-butanediamine (C4DA), and 1,4-benzenedithiol (BDT). A single interfacial configuration was observed for both BDA and C4DA junctions, while three different interfacial arrangements were resolved for BDT. This multiplicity is due to different molecular adsorption sites on the Au surface namely on-top, hollow, and bridge. Furthermore, C4DA junctions present a fluctuating *I*-*V* curve arising from the greater conformational freedom of the saturated alkyl chain, in sharp contrast with the rigid aromatic backbone of both BDA and BDT.

Understanding charge transport through single molecules is a fundamental issue in molecular electronics^1^,^2^,^3^,^4^,^5^,^6^,^7^,^8^,^9^. In recent years, increasing experimental and theoretical efforts have been devoted to the electronic characterization of a wide variety of single molecules. According to current understanding, structural details at the metal-molecule interface play a critical role in charge transport across a single-molecule junction. For example, the electronic conductance of the single-molecule junction is sensitive to metal-molecule contact configurations and molecular conformations^10^,^11^,^12^,^13^,^14^. Therefore, to determine the single-molecule conductance, repeated formation and measurement of single-molecule junctions and statistical analyses of the individual molecular junctions have been routinely carried out using mechanically controllable break junction (MCBJ)^15^,^16^ and scanning tunneling microscopy-based break junction (STM-BJ) methods^17^. For example, Li *et al.* demonstrated that alkanedithiol-molecular junctions sandwiched by Au electrodes feature three distinct conductance states at a fixed bias voltage^11^. With the aid of theoretical calculations, these three states have been assigned to a single-molecule junction with different contact configurations and *trans*/*gauche* conformations of the alkanedithiol. For the majority of single-molecule junction studies, structural identification of junctions have been performed by combining measured conductance at a fixed bias voltage and theoretical support of transport calculations at the low bias limit. This is because in BJ experiments at room temperature, the lifetime of a single molecule trapped between two electrodes is relatively short. The short life time of single-molecule junctions (<100 ms^18^,^19^) makes it difficult to routinely perform spectroscopic measurements such as surface enhanced Raman scattering (SERS)^20^,^21^ and current-voltage (*I*-*V*) characteristics^4^,^22^,^23^,^24^,^25^,^26^. The *I*-*V* characteristics of single molecular junctions provide useful information on the molecule-metal contact configurations, such as the electronic coupling between the metal and the molecule (*Γ* ) and the energy difference between the Fermi level energy and the conduction-orbital (*ε*_0_)^27^,^28^,^29^,^30^. Despite the wealth of information contained in the *I*-*V* characteristics of single molecular junctions, these measurements still remain a challenge from both, the experimental point of view, and in terms of analysis and interpretation. For example, the influence of the voltage scan rate remains a matter of debate. Previous studies have typically employed *I*-*V* acquisition times of approximately 100 ms^22^,^26^. This time-span is comparable to the sub-second lifetimes of single-molecule junctions prepared in ambient conditions by means of STM-BJ. Hence, the reliability of the *I-V* measurements will certainly benefit from faster voltage scan rates. Furthermore, reducing the *I-V* acquisition times will reduce the structural instability caused by current-induced Joule heating effects^31^.

In this study, we developed a robust statistical approach to obtain a detailed description of the metal-molecule interface in single-molecule junctions based on *I-V* measurements based on the STM-BJ method (Fig. 1a,b). A custom-made dataflow program was employed to control the STM in a semi-automated fashion, enabling the routine collection of *I-V* characteristics with reduced acquisition times up to 2.5 ms. To that end, a triangular voltage pulse was introduced into an otherwise typical STM-BJ procedure to collect the *I*-*V* characteristics of every junction formed. These experiments were repeated until a statistically significant dataset was obtained. In addition to the standard analysis in the conductance (*G*), statistical analysis of the *I*-*V* curve provides *Γ* and *ε*_0_, essential parameters needed to understand the metal-molecule contact configurations in molecular junctions. Combined analysis in *G* and both *Γ* and *ε*_0_ enabled us to resolve the structural details of the metal-molecule interfaces at the molecular junctions. We applied this approach to the single-molecule junctions of 1,4-benzenediamine (BDA), 1,4-butanediamine (C4DA), and 1,4-benzenedithiol (BDT) (Fig. 1c). BDA and C4DA bind to the Au electrodes through the same functional groups, but have different molecular backbones; in contrast, BDA and BDT have the same rigid molecular backbone but different anchoring groups. Single-molecule junctions with NH_2_ anchoring groups have well-defined conductance values^32^. Therefore, we first investigated the BDA molecular junctions, and then, extended our study to C4DA molecular junctions to understand the effect of molecular conformation on the *I*-*V* characteristics. Finally we demonstrated that, for the prototypical BDT junction^16^,^33^,^34^,^35^ with a variety of metal-molecule contact configurations, our approach can capture not only electronic details but also resolve structural details in the molecular junctions based on statistical analysis in *Γ* and *ε*_0_ with the support of *ab initio* charge transport calculations.

## Results and Discussion

It proved difficult to take stable *I*-*V* measurements of the single-molecule junction at slow scan rates due to significant current fluctuation, most probably arising from variation in the metal-molecule contact configuration structure and molecular conformation. We checked bias-voltage-scan-rate dependence of the current fluctuation within the range of 4 to 400 Hz, where the current fluctuation displayed considerable scan-rate dependence for BDA molecular junctions (Figs S1 and S2). Because BDA contains a rigid benzene backbone, the current fluctuation is most probably due to effects arising from structural variations in the metal-molecule contact configuration. We found that a scan rate of 40 to 400 Hz was fast enough to obtain *I*-*V* curves without large current fluctuations.

Figure 2a shows an example of an *I*-*V* curve of the BDA molecular junction. Two dimensional (2D) *I*-*V* histograms (Fig. 2b) were constructed from 1,000 *I*-*V* curves measured for the BDA molecular junctions, in which clear two band structures of *I*-*V* curves are apparent. The two bundles of the *I*-*V* curves, those of preferential molecular (low and high) conductance states, appear as current peaks at 290 and 450 nA (0.013 and 0.020 *G*_0_, where *G*_0_ = 2*e*^2^/*h*) at 0.3 V in the current histogram (Fig. 2c). The lower conductance state with 0.013 *G*_0_ is in good agreement with the molecular conductance of 0.01 *G*_0_ reported in the literature^36^. The small difference in conductance values between 0.013 and 0.01 *G*_0_ can be explained by the difference in the experimental conditions, such as the applied bias voltage for the charge transport. On the basis of peak positions in the current histogram, the *I*-*V* curves passing through the two current windows of 240–370 nA and 370–600 nA at 0.3 V are separated and averaged into two *I*-*V* curves, which are indicated by black dotted curves in Fig. 2b.

Within the single channel transport model, the transmission probability of a single-molecule junction can be represented by


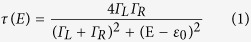


where *ε*_0_ and *Γ*_L(R)_ are the energy of the conduction channel (orbital) and the electronic coupling energy between the molecule and the left (right) electrode, respectively^23^,^33^,^35^. Here, we set the Fermi level, *E*_F_, to zero. The current through the molecular junction is written by


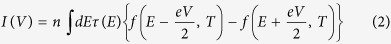


where *n* is the number of bridging molecules and *f* is the Fermi distribution function. When electronic temperature, *T*, is set to 0 K, Eq. 2 can be analytically evaluated as


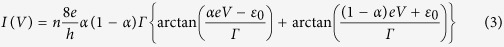


where *Γ* *=* *Γ*_L_ + *Γ*_R_ and *α* *=* *Γ*_L_/*Γ*_R_. Note that the temperature effect of the Fermi-Dirac distribution is several percent of *I*(*V*) at 300 K^35^.

Curve fitting the bias of Eq. 3 for the two preferential conductance states (*i.e.*, the two averaged *I*-*V* curves in Fig. 2b) reveals that the high and low conductance states correspond to a single conductance with a different number of *n* (*i.e.*, the low state; *n* = 1, *Γ* = 85 meV, and *ε*_0_ = 0.68 eV and the high state; *n* = 2, *Γ* = 75 meV, and *ε*_0_ = 0.71 eV). For the BDA molecular junctions, the “single” conductance state and the corresponding set of *Γ* and *ε*_0_ values were obtained by fitting statistically averaged *I*-*V* characteristics within a reasonable choice of *n* (See Table 1 and Supplementary Information, Section 2 for a further detail), which indicates that the single BDA junction displays a single conductance state with a preferential metal-molecule contact configuration^12^,^19^,^32^, Such preferential “NH_2_-Au” bonding and corresponding contact configuration has been revealed in the previous conductance measurements in combination with DFT-based transport calculations^37^; in these, a BDA molecule is in contact with an undercoordinated Au atom (*i.e*., the on-top site).The “Au–NH_2_” contact configuration originates from a simple delocalization of the lone pair of electrons from the amine-nitrogen to the Au atoms, and the bonding is not strongly directional^32^. Therefore, the electronic properties of the BDA junction is relatively insensitive to the molecular orientation on the Au electrode.

To clarify the effect of molecular backbone on the molecular *I*-*V* characteristics, we focused on C4DA, which has the same binding group, “-NH_2_”, as BDA but has a flexible molecular backbone that can adopt *trans* and *gauche* conformations^11^,^13^. In contrast to the rigid benzene backbone in BDA, C4DA is subject to conformational fluctuations in addition to bonding fluctuations (*i.e.*, structural changes in the metal-molecule contact configuration). We extended our *I*-*V* measurement technique to C4DA molecular junctions. Figure 3a shows a typical *I*-*V* curve of the C4DA single-molecule junction, in which the significant current fluctuation, most probably due to the flexibility of the methylene backbone, is apparent^22^. Figure 3b shows a 2D *I*-*V* histogram constructed from 1,000 *I*-*V* curves for the C4DA molecular junctions. The histograms exhibit several faint band structures, which can be explained by formation of preferential conformers with *trans* and *gauche* conformations at the C4DA molecular junctions. Here, we focus on the one of the bands, whose current ranged between 15 and 25 nA at 0.3 V, covering the previously reported conductance values of single C4DA molecule junctions measured by STM-BJ^32^. In a similar manner as in the BDA-*I*-*V* measurement, the curves within the current window have been averaged, and these are indicated by the black line shown in Fig. 3b. The linear shape of the averaged *I*-*V* curve of the C4DA molecular junctions is remarkably dissimilar to the nonlinear curves of the BDA molecular junctions. To qualitatively discriminate the *I*-*V* characteristics and related electronic structures between the molecular junctions, we fitted the averaged C4DA-*I*-*V* curves with eq. 3 under a constraint condition of *n* = 1. A set of *Γ* and *ε*_0_ values was determined to be *Γ* = 48 meV and *ε*_0_ = 1.7 eV. The obtained *ε*_0_ of 1.7 eV is substantially larger than the *ε*_0_ of 0.7 V obtained for the BDA molecular junction. This remarkably large energy of 1.7 eV is caused by intrinsic molecular nature in the junctions, which is a wide HOMO-LUMO gap of the insulating alkane moiety in the C4DA junction. Statistical analysis of the *I*-*V* curves enabled us to capture molecular dependent *I*-*V* characteristics in a qualitative manner, which was used to assess the applicability of the present method. It is interesting to note that, despite both of the C4DA and BDA junctions have the same Au-N binding group, much lower electronic coupling strength was found for C4DA (48 meV) than BDA (75~85 meV). The electronic coupling strength depends on not only the local structure of the binding group but also electronic details in the molecular backbones (*e.g.*, orbital delocalization in the molecular junction). The terminal N atom in BDA binds to a *sp*^2^-hybridezed carbon atom and the lone pair in the N atom is partly delocalized into the π-electron network in the molecular backbone. The resulting electronic interaction between the terminal binding group of N and the molecular backbone results in the larger coupling strength. On the other hand, the N atom in C4DA binds to a *sp*^3^-hybridized C atom and electronic interaction through the lone-pair in the N atom and the molecular backbone cannot be expected for C4DA. Thus, the electronic coupling of the molecular junction becomes smaller for C4DA. Such electronic interaction between the binding group and the molecular backbone has been demonstrated in rupture force measurements of BDA and C4DA molecular junctions sandwiched by Au electrodes, in which the BDA and C4DA junctions with the same Au-N binding group exhibited distinct rupture forces of the molecular junctions^38^.

To allow characterization of the metal-molecule interface of single-molecule junctions, we applied our statistical *I*-*V* analysis to BDT molecular junctions. Since the pioneering MCBJ-*I*-*V* experiments of Reed *et al.*, BDT is well-known as a prototypical molecule for electronic transport study of single-molecule junctions in the field of molecular electronics. Over the last decade, much research has been carried out, and a wealth of information on the variable transport properties of single molecule junctions has been accumulated, allowing a better understanding of the atomistic details at the junctions. The current understanding is that different “S-Au” bonding patterns, such as on-top, bridge and hollow adsorption-modes, and BDT-metal contact configurations at the molecular junctions, which have been demonstrated to display a variety of configurations, lead to differences in molecular conductance. One of the most challenging tasks is to identify the contact configurations and reliably characterize the corresponding charge transport properties and electronic structures at the molecular junctions. Figures S4, 4a,b show the typical *I*-*V* curves and 2D *I*-*V* histograms in two different current regimes. In contrast to the BDA junctions with a single “N-Au” bonding mode, the BDT junctions with multiple “S-Au” bonding modes features a variety of statistically significant *I*-*V* characteristics, as shown in Fig. 4a,b. To capture these complicated *I*-*V* characteristics, we developed an automated algorithm to fit each *I*-*V* curve with eq. 3 and extract the values of *Γ* and *ε*_0_. Figure 4c,d show 2D histograms of the fitted *Γ* and *ε*_0_ values constructed from 1,000 individual *I*-*V* curves measured for the BDT molecular junctions; in these, three preferential distributions, H, M, and L, are noticeable. The histograms of each *Γ* and *ε*_0_ (Fig. S5) value confirmed that there are preferential peaks-values for *Γ* = 31 and 126 meV and *ε*_0_ = 0.63 eV for *I*-*V*s in the large current regime (Fig. 4a) and peak values of *Γ* = 12 meV, and *ε*_0_ = 0.65 eV for *I*-*V*s in the small current regime (Fig. 4b). Curve-fitting and statistical analysis of the individual *I*-*V*s revealed the existence of three preferential conductance states, H, M, and L, which can be recognized as band structures in the 2D *I*-*V* histograms (Fig. 4a,b). The current histograms at the bias voltage of 0.3 V revealed one current-peak at 7 nA in the small conductance regime (Fig. 4f) and three current-peaks at 20, 60, and 670 nA in the large conductance regime (Fig. 4e), which correspond to 0.3, 0.9, 2.6, and 29 m*G*_0_ in order of conductance. A closer examination of the 2D *I*-*V* histograms revealed that additional fine structure appears in the large conductance regime, in which the M state is likely to split into two states. Based on the peak-positons in the current histograms, the *I*-*V* curves passing through the current windows of (L) 5~20, (M1) 20~40, (M2) 40~100 nA, and (H) 440~2200 nA at 0.3 V are divided into four groups to obtain statistically significant *I*-*V* characteristics. Averaged *I*-*V* curves within the windows are indicated by black dotted curves in Fig. 4a,b. Considering the number of bridging molecules (for further detail see Supplementary Information, Section 6 and Fig. S6), three preferential conductance states were identified for the BDT molecular junctions. By fitting statistically averaged *I*-*V*s of each using eq. 2, *Γ*, *ε*_0_, and *α* were determined for the H, M, and L states (Table 1), which originate from distinct “Au-S” contact configurations in the BDT molecular junctions.

To justify our experimental analysis and to assign the three conductance states, H, M, and L, to contact configurations of BDT, we calculated the *I-V* characteristics and properties of conductive molecular orbital (MO) for several model systems using nonequilibrium Green’s function combined with DFT (NEGF-DFT)^39^,^40^,^41^. We examined the three possible anchoring positions, *i.e.*, hollow, bridge, and on-top, to the Au electrodes and determined the conformations of each junction model by standard DFT geometry optimization. The conductance values of hollow and on-top conformations were 0.024 *G*_0_ and 0.009 *G*_0_, respectively. On the bridge type configurations, we found slightly different three stable structures, which are termed as (i) bridge, (ii) bridge-top, and (iii) tilted bridge. In the case of bridge-top, one anchoring point is the bridge site and the other site shifted slightly to the on-top position from the bridge site. The tilted bridge configuration is that the BDT molecule tilts by anchoring nonequivalent bridge sites of left and right electrodes. The schematic figures of these structures are given in Figs S9 and S10. Although the detailed structures of these bridge type conformations are different, all of the conformations have high conductance, *e.g.*, (i) bridge, 0.22 *G*_0_; (ii) bridge-top, 0.32 *G*_0_; and (ii) bridge tilt, 0.27 *G*_0_, respectively (Table S3). The conductance of the bridge “family” is much higher than that those of the hollow or atop configurations. In Fig. 5, the structural models and the calculated *I-V* curves of on-top, hollow, and bridge configurations are plotted. From these results, we conclude that our calculations also show three distinct conductance regimes for BDT, and they can be assigned by anchoring sites, *i.e.*, H is bridge, M is hollow, and L is on-top, respectively (Table 2). As described below, we chose the most conductive bridge type configuration, bridge-top, as the H state for further analysis.

Next, we discuss the relationship of the assigned sites and *Γ*, obtained by fitting the experimentally observed *I-V* using eq. 2. We define the projected molecular orbital (PMO) by diagonalizing the molecular projected Hamiltonian (MPSH) and identify the conductive MO, whose energy *ε*_a_ should be close to *E*_*F*_ and whose coupling strength to electronic state of the electrodes, *γ*, is sufficiently large^42^,^43^. The value of *γ* is the imaginary part of the normalized self-energy to MPSH and was obtained for each PMO. Generally, the conductive MO is not the conduction channel state^43^. Thus (*ε*_a_, *γ*) is not equal to (*ε*_0_, *Γ* ), as defined by eq. 2. However, identifying conducting MO’s is useful to check validity of our analysis *via* eq. 1. In addition, (*ε*_a_,*γ*) is a good approximation that allows discussion of the tendency of *γ* as far as we can select suitable conductive MOs. When we rewrite (*ε*_a_,*γ*) of the conductive MO’s of the H state as (*ε*_H_,*γ*_H_) *etc.*, the calculated values *ε*_H_, *ε*_M_, and *ε*_L_ are −0.75, −1.09, and −0.47 eV, respectively. Since the conductive MO energies of the three states are of the same order, analysis of the relationship between the contact configuration and *Γ*, as evaluated by *I-V* curves using eq. 1, is reasonable. The relative coupling strength 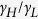
and 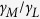
are 64 and 50, *i.e.*, the correlation of conductance and *Γ* agree reasonably with that of conductance and *γ*.

Towards the characterization of metal-molecule interface of single-molecule junctions, we developed a statistical approach for treatment of *I*-*V* measurements and analysis of single-molecule junctions measured by STM-BJ method under ambient conditions. For BDA, C4DA, and BDT molecules that are commonly used in the break junction-based-conductance measurements at a fixed bias voltage, we applied our statistical *I*-*V*-approach to determine the molecule dependent properties *Γ* and *ε*_0_ in a qualitative manner within the single channel transport model. For BDA and C4DA, the molecule dependent *ε*_0_ of 0.7 and 1.7 eV were obtained, which assessed applicability of the statistical *I*-*V*-approach. For BDT with a variety of metal-molecule contact configuration, three sets of statistically significant *I*-*V* characteristics with remarkable difference in the *Γ* values were captured and, by combining the result of first principle charge transport calculations, we identified the BDT junction-structures with on-top, hollow, and bridge sites-adsorption-configurations in the order of the molecular conductance.

## Methods

### *I*-*V* measurement of the molecular junctions

**Figure 1:**
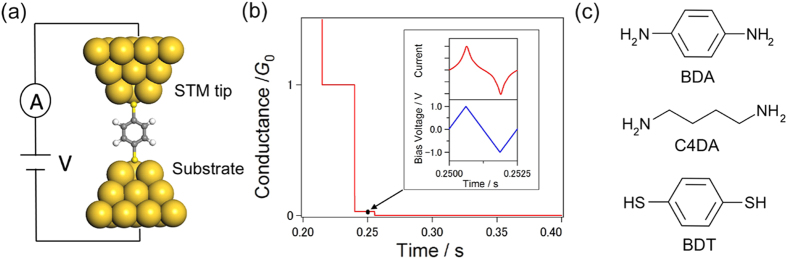
(**a**) Schematic illustration of STM-BJ setup. White, grey, yellow, and orange balls correspond to H, C, S, and Au atoms, respectively. (**b**) Schematic illustration for conductance (current) change as a function of time in one cycle of the *I*-*V* measurement. After making the metal contact, the contact was stretched at bias voltage of 20 mV. When the current drops below 2 μA, the Au tip position was fixed. Then the bias voltage was scanned from 20 mV to 1000 mV, –1000 mV, and back to 20 mV to measure the *I*-*V* curve of the single-molecule junction (see inset). Finally, the Au tip is pulled further away from the surface to break the molecular junction. (**c**) Molecular structures of 1,4-benzenediamine (BDA), 1,4-butanediamine (C4DA), and 1,4-benzenedithiol (BDT).

**Figure 2:**
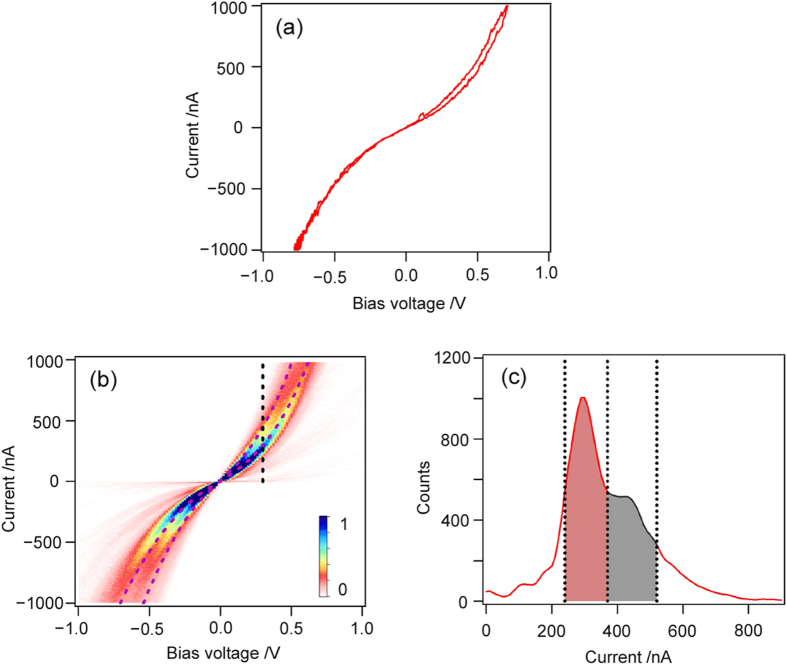
(**a**) Example of an *I*-*V* curve of the BDA molecular junction recorded at forward and backward bias-voltage scan (scan rate = 40 Hz). (**b**) Two dimensional (2D) *I*-*V* histogram of the BDA molecular junction, constructed from 1,000 *I*-*V* curves. Bin size is 0.016 V × 10 nA. The color scale shows the normalized current values. Dotted curves are averaged *I*-*V* curves within the current windows of 20~370 nA and 370~520 nA at 0.3 V. The two windows correspond to the red and black colored areas in (**c**). (**c**) Current histogram of BDA molecular junction at the bias voltage of 0.3 V (see the dotted line in (**b**)). Bin size is 10 nA.

**Figure 3:**
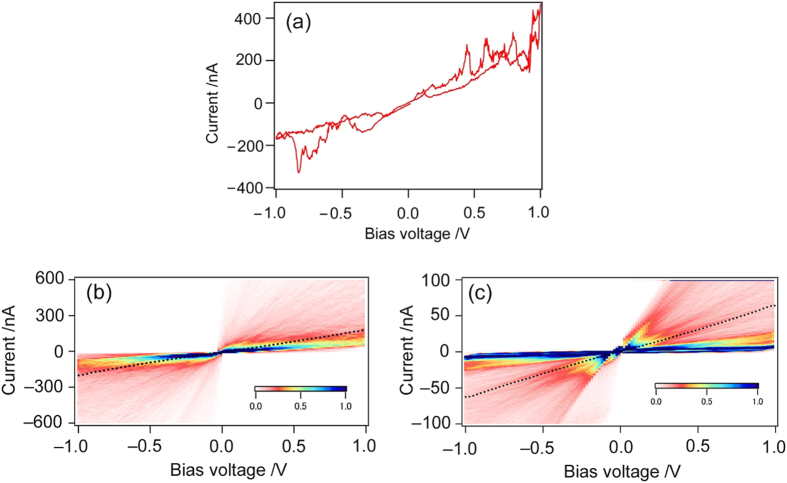
(**a**) Example of *I*-*V* curves for the C4DA molecular junction measured for both forward and backward bias voltage scans (scan rate = 400 Hz). (**b**) 2D *I*-*V* histogram of the C4DA molecular junctions constructed from 1,000 *I*-*V* curves. The current regime was 0–800 nA (**c**) Magnified view of (**b**) in the current regime between 0 and 100 nA. Bin sizes are (**b**) 0.016 V × 10 nA and (**c**) 0.016 V × 1 nA. Dotted lines in (**b**,**c**) are the averaged *I*-*V* curves within the current window of 15–25 nA at 0.3 V.

**Figure 4:**
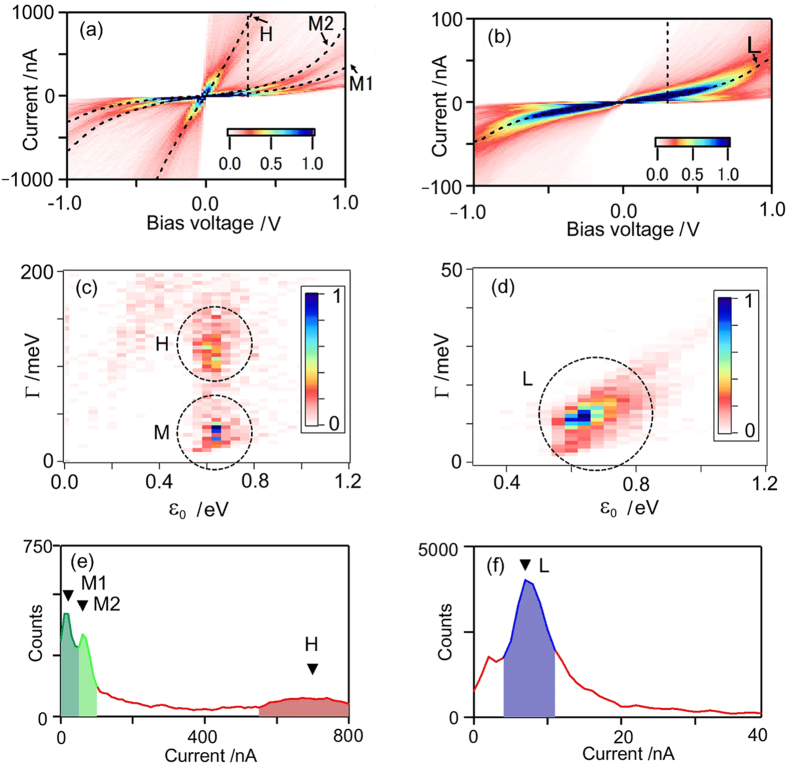
(**a,b**) 2D *I*-*V* histograms of BDT molecular junctions constructed from 1,000 *I*-*V* curves (Bias voltage scan rate = 400 Hz) measured by the two different current amplifiers ((**a**) 1 μA/V and (**b**) 10 nA/V) in separate experiments. Bin sizes are (**a**) 0.016 V × 10 nA and (**b**) 0.016 V × 1 nA. Dotted lines indicate bias voltage of 0.3 V. Dotted curves are the averaged *I*-*V* curves of the junctions within current windows of 4–11 in (**b**), and 0–50, 50–100, and 540–1300 nA in (**a**) at 0.3 V. The averaged *I*-*V* curves are denoted L, M1, M2, and H in order of the molecular conductance. See also (**e,f**). (**c,d**) 2D histograms of a set of the *Γ* and *ε*_0_, which were obtained by fitting each 1,000 *I*-*V* curves using eq. 3. The bin sizes are 0.04 eV. The same data set is used in (**a**–**d**). Three preferential distributions of H, M, and L are marked by dotted circles. (**e,f**) Current histograms of the BDT molecular junctions at the bias of 0.3 V. See the dotted line in (**a,b**). The peak position of three states, H, M1, and M2, are indicated by arrows (See also Table S1). The current windows for the *I*-*V* averaging in (**a,b**) were chosen based on the peak-currents and indicted by shaded areas in the current histograms.

**Figure 5:**
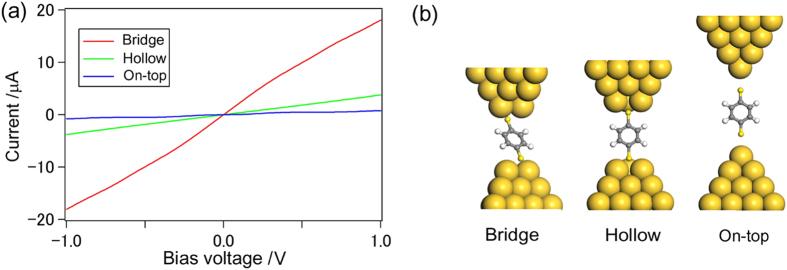
Theoretical *I*-*V* curves of BDT junctions for the bridge, hollow, and on-top adsorption-configurations, which are represented by red, green, and blue lines, respectively.

BDA, C4DA, and BDT were purchased from TCI Japan (Fig. 1c) and were used without further purification. The Au(111) substrate was prepared by thermal deposition of gold on mica at elevated temperature under high vacuum^44^. The sample for the *I*-*V* measurement was prepared by dipping the Au substrate into a 1 mM ethanol solution containing the target molecules. After evaporation of the solution, the substrate surface was washed with ethanol. We used a commercially available STM (Nanoscope V, Bruker, Santa Barbara, CA) operating at ambient conditions. Two current amplifiers, 1 μA/V and 10 nA/V, were used to access wide molecular conductance ranges from 10^–5^ to 10^1^ *G*_0_. STM tips were prepared by mechanically cutting an Au wire (Nilaco, diameter ≈ 0.3 mm, purity >99%). The *I*-*V* curves of the single-molecule junction were obtained by the following procedure (Fig. 1a,b). Firstly, an Au point contact (~10 *G*_0_) was made between the STM tip and the sample surface. Secondly, the tip was withdrawn by 10 nm at a speed of 38 nm/s to break the Au contact and to make a nanogap between the Au electrodes, forming the molecular junction during current monitoring at a fixed bias voltage of 20 mV. Thirdly, the tip position was fixed and one *I*-*V* curve was recorded by scanning the bias voltage from 20 to 1000, –1000 mV, and back to 20 mV within a time period of 2.5~25 ms at constant tip-sample separation. Finally, the junction was broken by pulling the STM tip away from the substrate. To capture possible structural variation of the junction structures, we cycled the molecular junction making and breaking process and reformed the junction-structure after obtaining each *I*-*V* curve. This *I*-*V* measurement-scheme was performed though a signal access module III (Bruker, Santa Barbara, CA) using an external piezo driver (E-665 LVPZT-Amplifier, Physik Instrumente) and a data-acquisition-device with LabVIEW2014 (USB-6363, National Instruments). More than 1,000 *I*-*V* curves for the molecular junctions were collected for each molecule. The *I*-*V* curves of molecular junction were obtained by automatically removing *I*-*V* curves corresponding to Au-metallic junctions and vacuum gap formation. For the *I*-*V* measurement using the 1 μA/V (10 nA/V) current amplifier, *I*-*V* curves with <100 nA (<5 nA) current at the bias of 1.0 V was classified as vacuum tunneling, while *I*-*V* curves with <10,000 nA (<100 nA) current at the bias of 0.2 V was classified as charge transport through an Au-metallic contact.

### Theoretical calculations

In this section, we survey the computational details used in the present first-principles calculations. The adsorption structures of BDT on Au electrodes were determined using a cluster model, where each side of the junction consists of 19 Au atoms and the cluster structure is taken to model the apex and the (111) surface of bulk electrodes. We fixed the two outermost Au layers with the structure of the bulk (clipped bulk), and the other atomic positions and the distance between the distance of the left and right electrodes were allowed to relax. We took hollow, bridge, and top adsorption sites as initial geometries and examined the tilt adsorption structure, *i.e*., the alignment of S-S axis of BDT molecule tilts to the surface. We used density functional theory (DFT) to carry out the calculations and the B3LYP exchange-correlation (XC) functional and LanL2DZP basis set for cluster model calculations. For the DFT calculations, Gaussian09 was used. To carry out transport calculations, we replaced the clipped bulk part of the cluster model with a *c*(5 × 5) bulk model by adding shortage Au atoms and then adding three more Au atomic layers to set up a scattering region. A periodic boundary condition was used, thus the unit cell was defined by a *c*(5 × 5) structure. We used the Perdew–Burke–Ernzerhof (PBE) XC functional for NEGF-DFT calculations. For the NEGF-DFT calculations, double-zeta plus polarization function basis set for all atoms in the molecule and a single-zeta plus polarization function basis set for the Au atoms were used. To check the validity of the evaluated conductance values and analysis, we also examined the XC using the local density approximation self-interaction correction (LDA-SIC). We confirmed that the PBE functional provides sufficient results for qualitative analysis in the present purpose, *i.e.*, evaluation of the electronic coupling strength and identification of each conductance state (H, M, L). All the NEGF-DFT calculations were performed using the HiRUNE subroutine^39^ and Smeagol^40^,^41^, which are both interfaced with the SIESTA package^45^. The electronic coupling strength and energy level of conductive molecular orbital (MO), which is qualitatively related to *Γ* and *ε*_0_, were evaluated directly by calculating projected MO’s (PMO) and renormalizing the self-energy of each PMO. The details of the method, which is called the effective molecular projected state Hamiltonian (MPSH) approach, is given in ref. [42].

## Additional Information

**How to cite this article**: Komoto, Y. *et al.* Resolving metal-molecule interfaces at single-molecule junctions. *Sci. Rep.* **6**, 26606; doi: 10.1038/srep26606 (2016).

## Supplementary Material

Supplementary Information

## Figures and Tables

**Table 1:** List of *Γ*, *ε*_0_, *α* and conductance values for single BDT molecular junctions with three distinct conductance states (H, M, and L).

		*Γ*( = *Γ*_L_ + *Γ*_R_)/meV	*ε*_0_/eV	*α* *=* *Γ*_L_/*Γ*_R_	Conductance/m*G*_0_	
**BDA**	**L**	85	0.68	0.53	13	(*G*_BDA-L_)
	**H**(*n* = 1)	105	0.70	0.53	20	(1.5 *G*_BDA-L_)
	**H**(*n* = 2)≈**L**	75	0.71	0.53	10	(0.8 *G*_BDA-L_)
**C4DA**		48	1.70	0.52	0.8	
**BDT**	**L**	12	0.67	0.51	0.31	(*G*_BDT-L_)
	**M**	26	0.63	0.51	1.7	(5.5 *G*_BDT_-_L_)
	**H**	129	0.66	0.51	37	(119.4 *G*_BDT_-_L_)

*Γ*, *ε*_0_ and *α* were obtained by fitting the averaged *I*-*V* curves with **Eq. 3**. It should be noted that *α* = 0.5 was found for all the molecular junctions studied here. For a further detail, see Fig. S8.

**Table 2:** List of the calculated conductance and *ε*_0_ of the BDT junctions with the bridge, hollow, and on-top adsorption-configurations.

	*ε*_0_/eV	Conductance/m*G*_0_	
**BDT on-top**	–0.47	9	(*G*_on-top_)
**BDT hollow**	–1.09	24	(2.7 *G*_on-top_)
**BDT bridge**	–0.75	220	(24.4 *G*_on-top_)
