# Supplementary material for: Resolving metal-molecule interfaces at single-molecule junctions
Source: Sci Rep. 2016 May 25;6:26606. doi: 10.1038/srep26606 (PMC4879565; doi:10.1038/srep26606)
Supplement: Supplementary Information [file srep26606-s1.pdf]

Supplementary Information

Resolving metal-molecule interfaces at single-molecule junctions

Yuki Komoto[†], Shintaro Fujii*[†], Hisao Nakamura*[§], Tomofumi Tada[#], Tomoaki Nishino[†] and Manabu Kiguchi*[†]

[†]Department of Chemistry, Graduate School of Science and Engineering, Tokyo Institute of Technology O-okayama, Meguro-ku, Tokyo 152-8551, Japan

[§]National Institute of Advanced Industrial Science and Technology, Central 2, Umezono 1-1-1, Tsukuba, Ibaraki 305-8568, Japan

[#]Materials Research Center for Element Strategy, Tokyo Institute of Technology, 4259-S2-13 Nagatsuta-cho, Midori-ku, Yokohama 226-8503, Japan

Table of contents

1. Scan rate dependence of the *I*-*V* curves and current fluctuation analysis in the *I*-*V* curves
2. Curve-fitting of the averaged *I*-*V* curves for the BDA molecular junctions
3. Example of conductance traces for the BDA molecular junctions
4. Examples of the *I*-*V* curves for the BDT molecular junctions
5. Histograms of fitted values of ε_0 and Γ obtained from 1,000 of individual *I*-*V* curves of the BDT molecular junctions
6. Normalization of the averaged *I*-*V* curves for the BDT molecular junction
7. List of Γ, ε_0, α, and conductance values for the single BDT molecular junction with three distinct conductance states (H, M, and L)
8. STM-BJ measurements of the BDT molecular junction
9. Symmetry analysis of the shape of the *I*-*V* characteristics
10. Theoretical models
11. References

1. **Scan rate dependence of the *I*-*V* curves**

Figure S1 shows examples of *I*-*V* curves for the BDA molecular junctions measured for both forward and backward bias voltage scans. We checked bias-voltage-scan-rate dependence of the current fluctuation within the range of 4 to 400 Hz and found that, for BDA molecular junctions, the current fluctuation was considerably scan-rate dependent (Figure S1). Because the BDA molecule has rigid benzene backbone, the current fluctuation is most probably due to the effects of structural variation in the metal-molecule contact configuration. At slower scan rates, current fluctuation is apparent, which can be ascribed to current-induced heating and resulting structural changes in the metal-molecule contact configuration (see the upper panel in Figure S1). We found that a scan rate of between 40 and 400 Hz was fast enough to obtain *I*-*V* curves without the large current fluctuations most probably arising from structural changes in the metal-molecule contact configuration.

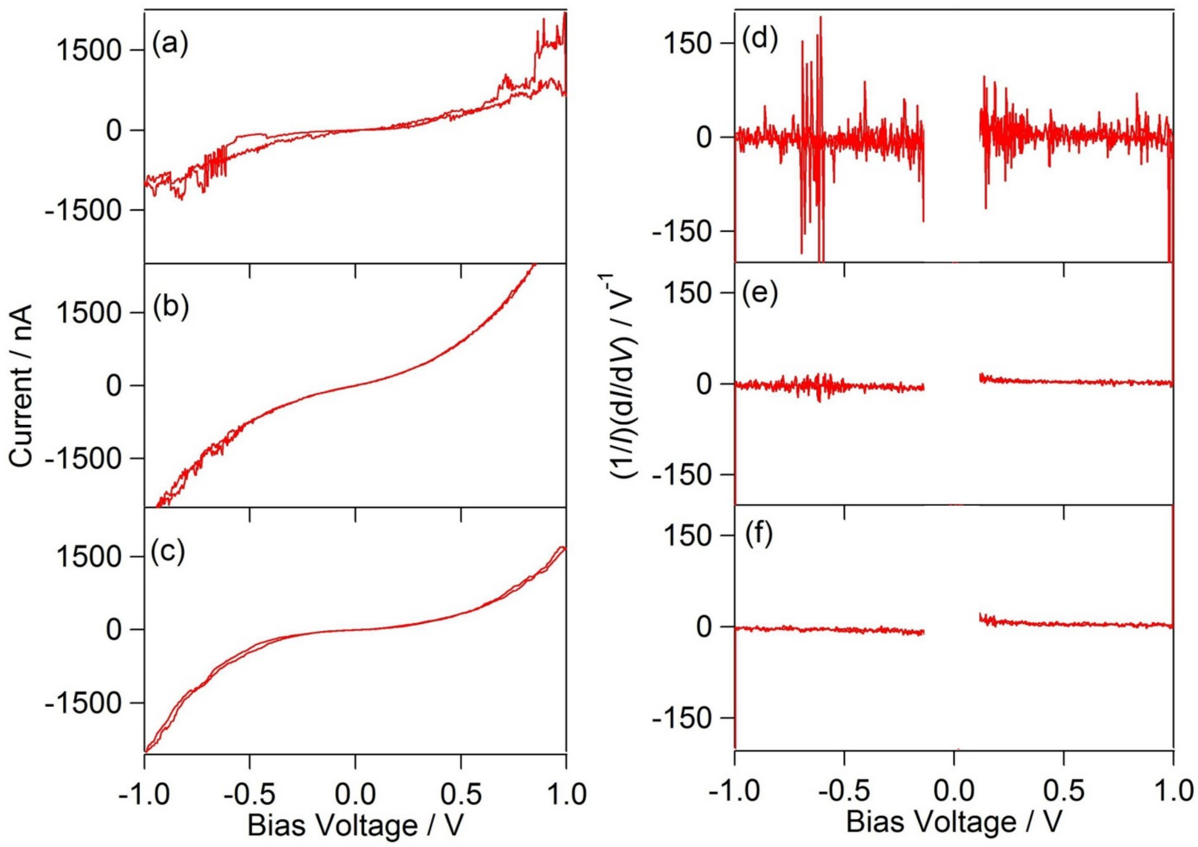

Figure S1. Examples of *I*-*V* curve and normalized (d*I*/d*V*)/*I*–*V* plot for the BDA molecular junction measured for both forward and backward bias voltage scans. The scan rate is (a,d) 4 Hz, (b,e) 40 Hz, and (c,f) 400 Hz. In (d)-(f), data in the lower bias range is not plotted due to inaccuracy in the bias voltage.

To statistically analyze the current fluctuation, we calculated the following quantity for each *I*-*V* curve.

$$\sigma = \frac{1}{n}\sum\frac{(I_{\text{measure}}-I_{\text{expect}})^2}{{I_{\text{measure}}}^2} \qquad \text{eq. 1}$$

where n is number of the current-data points in each *I*-*V* curve, I_{measure} is measured current at each data point, I_{expect} is expected current, which was calculated from linear fitting using 10 data points before the data point of I_{measure}. Figures S2a,b show histograms of the current fluctuation of σ (see eq. 1) for *I*-*V* curves with the scan rates of 40 Hz and 400 Hz for the BDA junction. At the lower scan rate of 40 Hz, the current fluctuation exhibits a peak value at 10^{-3}, while, at the faster scan rate at 400 Hz, the current fluctuation-peak shifted to lower value at $10^{-3.5}$. It should be noted that *I*-*V* curves were taken at the lower scan rate of 4 Hz but we could not constantly obtained *I*-*V* curves due to instability of junction structures, in which the junctions were broken during the *I*-*V* measurements.

Figures S2c,d shows histograms of the current fluctuation of σ for the *I*-*V* curves with the scan rate of 400 Hz at the high (Fig. 4a) and low (Fig. 4b) current regimes for the BDT junction. Both histograms display a similar trend found for the BDA junction, in which we can identify current-fluctuation-peaks at around 10^{-4}. For the fluctuation histogram at the high current regime, larger distributions are noticeable at higher current fluctuation above 10^{-2}. Closer examination of *I*-*V* curves with the larger current fluctuation from 10^{-2} to 10^{-1} revealed that the current fluctuation was partially originated from discrete transition of current values in *I*-*V* curves, which can be due to sudden change in the BDT junction (*i.e.*, change in the metal-molecule contact configurations). Based on the structural models (Fig. 5), the structural changes correspond to the change in the metal-molecule contact configurations between the hollow and bridge binding geometries. In contrast to the on-top binding geometry at a larger electrodes-separation, the hollow and bridge

binding geometries can formed at a similar and smaller electrodes-separations, which can enable the structural transition between the he hollow and bridge binding geometries.

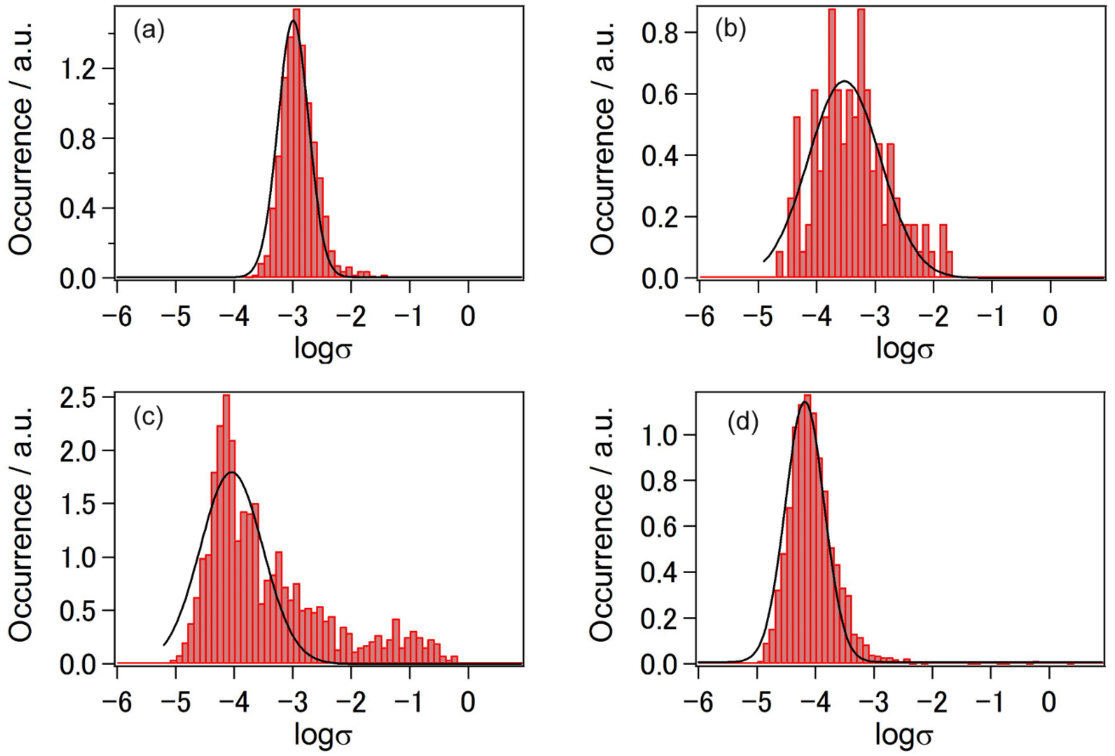

Figure S2. Histogram of the current fluctuation, logarithmic σ (see eq. 1), of *I*-*V* curves with the scan rates of (a) 40 Hz and (b) 400 Hz for the BDA junction and I-V curves of (c) high and (d) low current regimes for the BDT junction. Results of Gaussian fitting are indicated by black curves.

2. **Curve-fitting of the averaged *I-V* curves for the BDA molecular junctions**

Several studies have been conducted to develop statistical electronic characterization-methods based on the *I-V* characteristics of the single molecular junctions [S1-S4]. Lörtscher *et al.* reported statistical analysis of the *I-V* characteristics of single molecular junctions in which a dithiolated phenylene oligomer-derivative featured two sets of molecular *I-V* curves[S5]. Guo *et al.* demonstrated statistical analysis of transition voltage spectra (TVS) based on several thousand *I-V* characteristics for alkanedithiol-single molecular junctions[S6]. They found three metastable conductance states by statistically analyzing *I-V* curves and discussed the contact resistance based on the ε_0 obtained by TVS; however, they did not clearly mention the contact configurations. Adak *et al*. evaluated Γ and ε_0 for preferential single conductance states for pyridine-based molecular junctions on the basis of conductance measurement under bias-voltage modulation[S7]. Although there have been several studies of statistical *I-V* analyses that have clarified the existence of several preferential conductance states, the identification of the conductance states is still a challenging task, most probably due to difficulties in routinely performing repeated spectroscopic measurement of the single molecular junctions and analyzing the spectroscopic data in a qualitative manner.

In this study, we developed a statistical approach to measure the *I-V* characteristics of single molecular junctions in a qualitative manner using the single channel transport model and to identify the junction-structures on the basis of the *I-V* data. Curve fitting on the bias of **Eq. 3** for the two preferential conductance states of the BDT junction (*i.e.*, the two averaged *I-V* curves in Figure 2b) yielded values of Γ = 85 meV and ε_0 = 0.68 eV and Γ = 105 meV and ε_0 = 0.70 eV for low and high conductance states, respectively, in which the number of bridging molecules, *n*, was fixed to be 1. On the other hand, the high conductance state can be fitted by varying *n*, and we found a fitting result of Γ = 75 meV and ε_0 = 0.71 eV for *n* = 2 for the high conductance state (see also Table 1). The number *n* is likely to be arbitrary, but it has been reported in a large number of the previous single molecular conductance-studies that (i) the most probable number is 1, (ii) the maximum number of *n* is typically less than *n* = ~3 or 4, and (iii) formation probability of the multiple molecular junctions are low (*e.g.*, triple junction-formation probabilities of < 28%[S8], 18–55%[S9], and N.A.[S10] can be estimated by a comparison of molecular conductance-peak-intensities in conductance histograms. Therefore, the initial curve-fitting was performed with *n* = 1 and then the multiple-junction formation with *n*> 1 were considered in our

I-*V* fitting procedure). The obtained set of Γ and ε_0 values were closely similar to those found for the low conductance state. The obtained set of Γ and ε_0 is closely similar to that found for the low conductance state. For the BDA molecular junctions, the "single" conductance state and the corresponding set of Γ and ε_0 values were obtained by fitting statistically averaged *I*-*V* characteristics within a reasonable choice of n, which indicates that the single BDA junction displays a single conductance state with a preferential metal-molecule contact configuration.

It should be noted that, in a separate STM-BJ experiment at a fixed bias voltage, formation of the double BDA molecular junction with $n = 2$ can be seen, as shown in Figure S3.

3. **Example of conductance traces for the BDA molecular junctions**

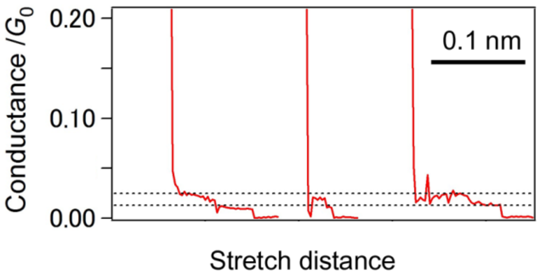

Figure S3. Conductance traces of the BDA junctions with double molecular junction formations. Black dotted lines represent the molecular conductance for the single (13 mG_0) and double (26 mG_0) junction-formation. Bias voltage was 20 mV. Tip velocity was 50 nm/s.

4. **Examples of the *I-V* curves for the BDT molecular junctions**

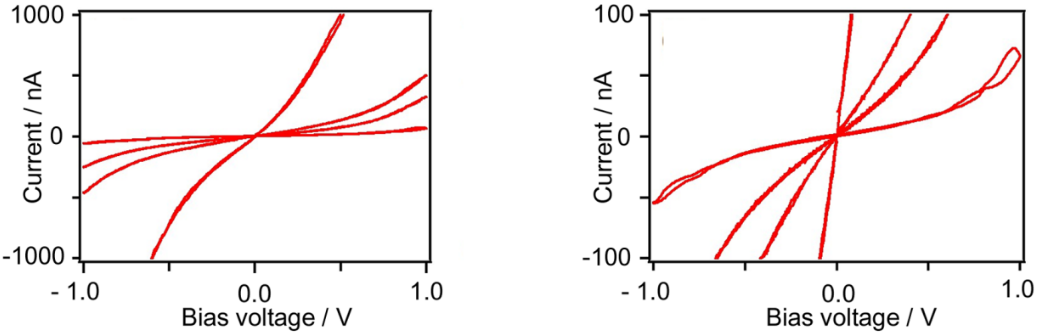

Figure S4. Example of *I-V* curves for the BDT molecular junctions measured at forward and backward bias voltage scans. Bias voltage scan rate was 400 Hz.

5. **Histograms of the fitted ε_0 and Γ obtained from 1000 of individual *I-V* curves of the BDT molecular junctions**

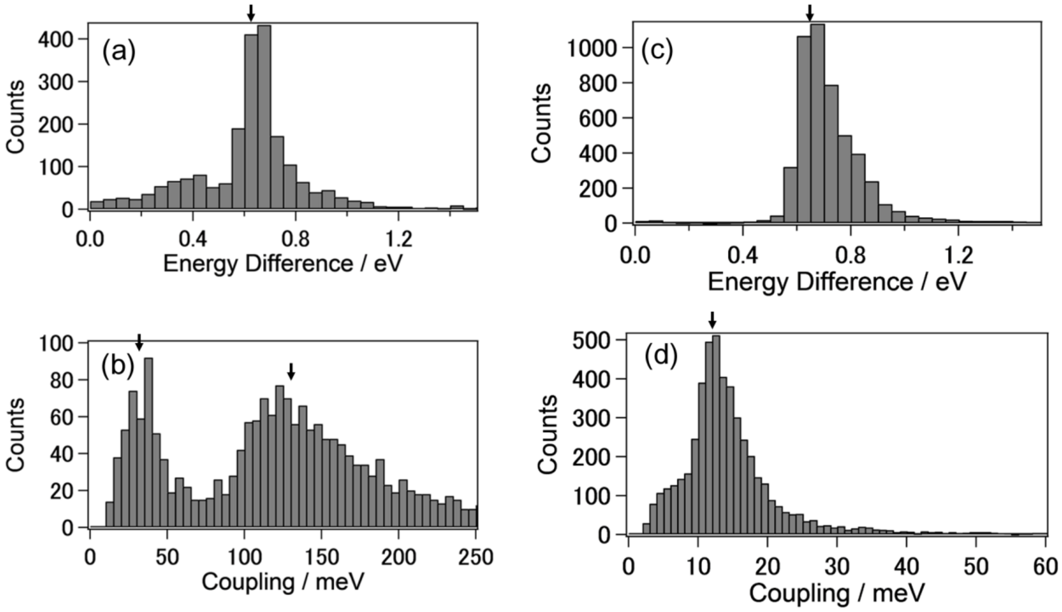

Figure S5. Histograms of the fitted ε_0 and Γ obtained from 1,000 of individual *I-V* curves of the BDT molecular junctions in the (a,b) large and (c,d) small conductance regime. Peak positions found by Gaussian fitting of the distributions are indicated by arrows; (a) 0.63 eV, (b) 31 meV and 126 meV, (c) 0.65 eV, and (d) 12 m eV.

6. **Normalization of the averaged *I*-V curves of the BDT molecular junction**

To take into account the number of bridging molecules (*n*), the statistically averaged *I*-*V* curves of M1, M2, and L were normalized to overlap them each other on the basis of the current at 0.3 V (Figure S6). The multiplication factors are 3, 1, and 0.5 for the curves of L, M1, and M2, respectively. The normalized curves of M1 and M2 are almost identical in the full bias-voltage arrangement, while there is a striking mismatch between L and M (M1 and M2). These results indicate that M1 and M2 belong to the same conductance state (*i.e.*, M) with different values of *n* = 1,2 (see Figure S6); furthermore, this reconfirms that M and L are distinct conductance states. It should be note here that the conductance of H is more than one order of magnitude larger than that of M, and therefore, the difference in the conductance between H and M is unlikely to be explained by the difference in the number of the bridging molecules (*n*).

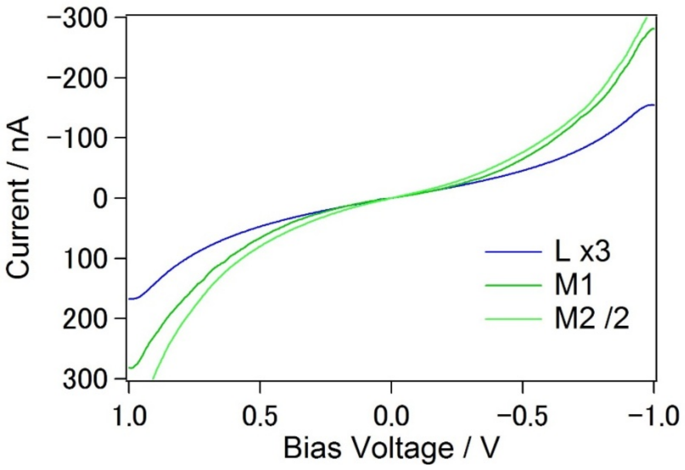

Figure S6. Averaged and current-normalized *I*-*V* curves of the BDT molecular junction at 0.3 V.

7. **List of Γ, ε_0, α, and conductance values of the single BDT molecular junction with three distinct conductance states (H, M, and L)**

		Γ(= $\Gamma_L + \Gamma_R$) / meV	ε_0 / eV	$\alpha = \Gamma_L / \Gamma_R$	Conductance / mG_0
**BDT**	**L**	13±3	0.70±0.09	0.51±0.03	0.3±0.1
	**M**	26±7	0.68±0.06	0.50±0.04	1.5±0.7
	**H**	132±59	0.69±0.28	0.51±0.02	36±9

Table S1. List of Γ, ε_0, α, and conductance values for the single BDT molecular junction with three distinct conductance states (H, M, L). Γ, ε_0, and α were obtained by the fitting 1000 of individual *I-V* curve using **Eq. 3**. The mean values and dispersions were calculated within the current (conductance) windows of the H, M, and L states, which were defined by the analysis in the 2D *I-V* histogram (see Figures 4e and 4f and the corresponding main text).

8. **STM-BJ measurement of the BDT molecular junction**

Figure S7 shows the conductance histogram for the single BDT molecular junction measured at the fixed bias voltage of 20 mV using the STM-BJ method. The histogram shows three peaks at 0.7, 2, and 20 mG_0, which correspond to the three states observed by the *I*-*V* measurement (see Table 1). The difference of the conductance value between the STM-BJ experiment and the *I*-*V* experiment can be explained by the experimental conditions. The conductance of the single molecular junction is measured during the stretching the contact at the fixed low bias voltage (20 mV) in STM-BJ experiment, while it is measured at the fixed electrode separation by sweeping the wide voltage-range (±1 V) for the *I*-*V* measurement.

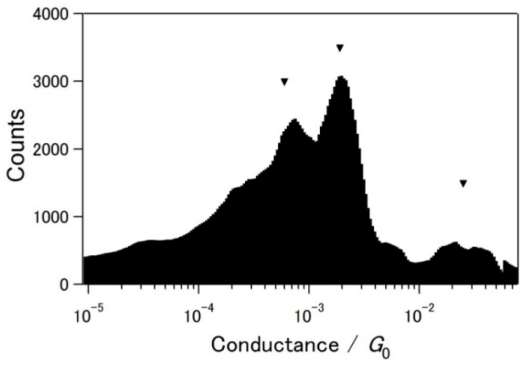

Figure S7. Conductance histogram of the single BDT molecular junction measured with the STM-BJ method. The histogram is constructed 200 conductance traces. Arrows indicated conductance peak positions.

State	Conductance / mG_0
**L**	0.7
**M**	2
**H**	20

Table S2. List of the BDT-single molecular conductance obtained by STM-BJ experiment.

9. Symmetry analysis in the shape of the *I-V* characteristics

Statistical analysis in the *I-V* characteristics of the single molecule junctions revealed that BDA and BDT studies here display symmetric shapes in the *I-V* characteristics with α = 0.5 ($\alpha = \Gamma_L / \Gamma_R$. See Eqs 1–3 and Tables 1 and S1). Figure S8 shows histograms of the current ratio in the molecular *I*-V curves at the bias voltages of ±1.0 V for the BDA and BDT molecular junctions. The current ratio was calculated by dividing the current at 1 V with that at –1 V in the *I-V* curves. The *I-V* curves were found to be symmetric at the positive and negative bias voltages with log(current ratio) = 0 (Figure S8).

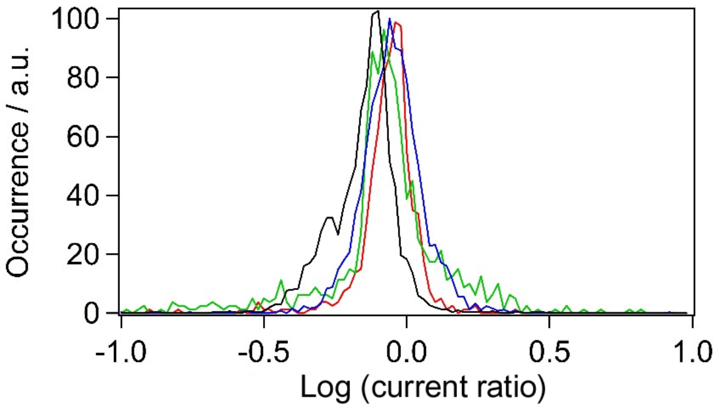

Figure S8. Distribution of the current ratio at the positive and negative bias voltage for the single molecular junction. Blue: L state of BDT, green: M state of BDT, red: H state of BDT, black: BDA. Bin size = log(R) = 0.1. The intensity was normalized by the number of samples.

10. Theoretical models

Figures S9 and S10 show theoretical models of BDT molecular junctions. In the present theoretical models of the BDT junctions, the Au electrodes (clusters) with different shapes consist of different numbers of Au atoms. Therefore, we could not directly compare the energetic stability of the calculated system. In previous theoretical studies, related energetic stability has been investigated for models with thiolate sitting on hollow, bridge and on-top sites on Au surface [S11-S13]. Although there are (small) discrepancy on the relative energetic stability among the theoretical calculations, the theoretical studies have suggested the existence of the hollow, bridge and on-top adsorption geometries for thiolate on Au surface. Based on the previous findings, the BDT-junction-models with the hollow, bridge and on-top adsorption-geometries were relaxed and used for the transport calculations in the present study. In addition to the above-mentioned Au/thiolate/Au junctions, we considered formation of Au/thiol/Au junctions, in which the S atom in the Au-S binding group is hydrogenated[S14,S15]. It should be noted that, although it has been commonly accepted that a thiol group becomes dehydrogenated during the adsorption and molecular film-formation process on Au surface[S16], theoretical studies have demonstrated existence of Au/thiol/Au junctions (*e.g.*, see refs [S11,S12]). In relaxed hydrogenated geometry (on-top geometry), molecular orbital that is responsible for charge transport displayed spatially localized nature, which leads to significant reduction of low bias molecular conductance.

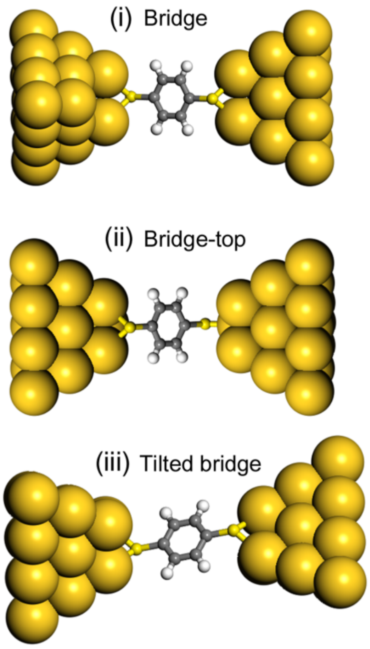

Figure S9. Schematic illustration of the bridge type configurations of the BDT molecular junctions; (i) bridge, (ii) bridge-top, and (iii) tilted bridge. White, grey, yellow, and orange balls represent H, C, S, and Au atoms.

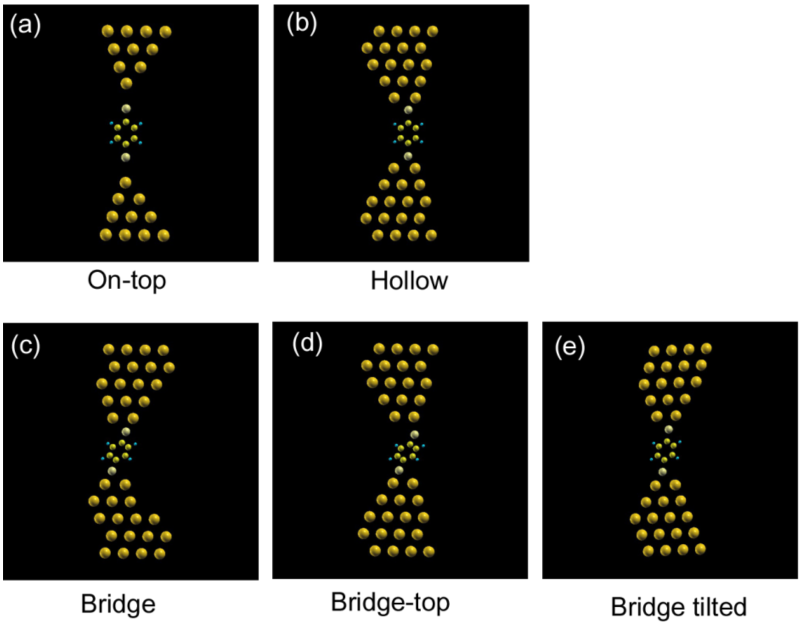

Figure S10. Structural models of (a) on-top, (b) hollow, (c) bridge, (d) bridge-top, and (e) bridge tilted configurations used for the DFT-transport calculations. Blue, yellow, pale yellow, and orange balls correspond to H, C, S, and Au atoms, respectively.

**Configuration**	**Conductance / mG_0**
**On-top**	9
**Hollow**	24
**Bridge**	220
**Bridge-on-top**	320
**Tilted bridge**	270

Table S3. List of the calculated zero bias conductance of on-top, hollow, bridge, bridge-top and tilted bridge configurations.

11. References

S1 Widawsky, J. R., Kamenetska, M., Klare, J. *et al.* Measurement of voltage-dependent electronic transport across amine-linked single-molecular-wire junctions. *Nanotechnology* **20**, 434009 (2009).

S2 Bruot, C., Hihath, J. & Tao, N. Mechanically controlled molecular orbital alignment in single molecule junctions. *Nat Nanotechnol* **7**, 35–40 (2012).

S3 Frisenda, R., Perrin, M. L., Valkenier, H., Hummelen, J. C. & van der Zant, H. S. J. Statistical analysis of single-molecule breaking traces. *Phys Status Solidi B* **250**, 2431-2436, doi:10.1002/pssb.201349236 (2013).

S4 Martin, C. A., Ding, D., van der Zant, H. S. J. & van Ruitenbeek, J. M. Lithographic mechanical break junctions for single-molecule measurements in vacuum: possibilities and limitations. *New J Phys* **10**, 065008 (2008).

S5 Lortscher, E., Weber, H. B. & Riel, H. Statistical approach to investigating transport through single molecules. *Phys Rev Lett* **98**, 176807 (2007).

S6 Guo, S., Hihath, J., Diez-Perez, I. & Tao, N. Measurement and statistical analysis of single-molecule current-voltage characteristics, transition voltage spectroscopy, and tunneling barrier height. *J Am Chem Soc* **133**, 19189–19197 (2011).

S7 Adak, O., Korytar, R., Joe, A. Y., Evers, F. & Venkataraman, L. Impact of Electrode Density of States on Transport through Pyridine-Linked Single Molecule Junctions. *Nano Lett* **15**, 3716-3722 (2015).

S8 Xu, B., Tao, N. J. Measurement of Single-Molecule Resistance by Repeated Formation of Molecular Junctions. *Science* **2003**, *301*, 1221–1223.

S9 Li, C., Pobelov, I., Wandlowski, T. *et al.* Charge Transport in Single Au | Alkanedithiol | Au Junctions: Coordination Geometries and Conformational Degrees of Freedom. *J. Am. Chem. Soc.* **2008**, *130*, 318–326.

S10 Venkataraman, L., Klare, J. E., Tam, I. W. *el al.* Single-Molecule Circuits with Well-Defined Molecular Conductance *Nano Lett.* **2006**, *6*, 458–462.

S11. Sellers, H., Ulman, A., Shnidman, Y. & Eilers, J. E. Structure and binding of alkanethiolates on gold and silver surfaces: implications for self-assembled monolayers. *J. Am. Chem. Soc.* **115**, 9389–9401 (1993).

S12. Vargas, M. C., Giannozzi, P., Selloni, A. & Scoles, G. Coverage-dependent adsorption of CH_3S and $(CH_3S)_2$ on Au(111): a Density functional theory study. *J. Phys. Chem. B* **105**, 9509–9513 (2001).

S13. Hayashi, T., Morikawa, Y. & Nozoye, H. Adsorption state of dimethyl disulfide on Au(111): Evidence for adsorption as thiolate at the bridge site. *J. Chem. Phys.* **114**, 7615–7621 (2001).

S14. Stokbro, K., Taylor, J., Brandbyge, M. Mozos, J.-L., & Ordejón, P. Theoretical study of the nonlinear conductance of di-thiol benzene coupled to Au(111) surfaces *via* thiol and thiolate bonds. *Comp. Mater. Sci.* **27**, 151–160 (2003).

S15. Tada, T., Kondo, M. & K. Yoshizawa, Green's function formalism coupled with Gaussian broadening of discrete states for quantum transport: Application to atomic and molecular wires. *J. Chem. Phys.* **121**, 8050–8057 (2004).

S16. Love, J. C., Estroff, L. A., Kriebel, J. K., Nuzzo, R. G. & Whitesides, G. M. Self-assembled monolayers of thiolates on metals as a form of nanotechnology. *Chem. Rev.* **105**, 1103−1169 (2005).
